# Grape VOCs Response to Postharvest Short-Term Ozone Treatments

**DOI:** 10.3389/fpls.2018.01826

**Published:** 2018-12-11

**Authors:** Susana Río Segade, Mar Vilanova, Matteo Pollon, Simone Giacosa, Fabrizio Torchio, Luca Rolle

**Affiliations:** ^1^Dipartimento di Scienze Agrarie, Forestali e Alimentari, Università di Torino, Turin, Italy; ^2^Misión Biológica de Galicia – Consejo Superior de Investigaciones Científicas, Pontevedra, Spain; ^3^Dipartimento di Scienze e Tecnologie Alimentari per una filiera agro-alimentare Sostenibile, Università Cattolica del Sacro Cuore, Piacenza, Italy

**Keywords:** ozone, postharvest treatment, partial dehydration, volatile compounds, terpenes, aromatic winegrapes

## Abstract

Ozone has been recently recognized as an efficient sanitizing agent in wine industry because of its powerful oxidizing properties. Furthermore, postharvest treatments of grapes with ozone can stimulate defense responses by synthetizing secondary metabolites against oxidative stress. In this study, the effect of postharvest short-term ozone treatments was assessed for the first time on free and glycosylated volatile organic compounds (VOCs) of winegrapes. Two different ozone concentrations (30 and 60 μL/L) and exposure times (24 and 48 h) were investigated just after treatment (fresh grapes) and after partial dehydration up to 20% weight loss (withered grapes). The study was carried out on Moscato bianco winegrapes (*Vitis vinifera* L.) due to the importance of terpenes in white aromatic cultivars to produce high quality wines. The results obtained showed that short-term ozone treatment caused a significant decrease in total contents of free VOCs in fresh grapes, mainly due to terpenes. Among them, linalool, geraniol, and nerol are the major aromatic markers of Moscato bianco grapes. Ozone entailed a significant decrease of free linalool contents in fresh grapes, the less stressful ozone treatment showing the smaller linalool degradation. However, the stronger and longer ozone treatment induced the synthesis of this compound probably in response to higher abiotic stress. Instead, significant changes were not observed in geraniol and nerol contents in fresh grapes. This last ozone treatment also reduced the loss of free linalool by water loss in withered grapes even though total VOCs and terpenes remained relatively stable. Furthermore, ozone seems to promote the synthesis of free (+)-4-carene and 4-terpineol in withered grapes under certain treatment conditions. Regarding glycosylated compounds, total VOCs and terpenes were less sensitive to ozone. Our findings highlight that ozone can be used as sanitizing agent in aromatic grape varieties prior to winemaking without affecting sharply the aromatic profile of fresh grapes and even improving it in withered grapes.

## Introduction

After harvest, fruits remain metabolically active and are subjected to continuous physical and chemical changes, including degradation and/or biosynthesis reactions. It is well known that the fruits react to internal and external stimuli and stresses both in vineyard and postharvest, through a chemical defense response affecting their composition (Ferrandino and Lovisolo, 2014; Carbone and Mencarelli, 2015). However, these compositional changes can be modulated by postharvest controlled stresses to increase the phytochemical content of fruits (Schreiner and Huyskens-Keil, 2006). Particularly in grape berries, the quality is mainly dependent on secondary metabolites, primarily phenolic and volatile compounds. Biotic and abiotic stresses can be exploited to stimulate the synthesis of these secondary metabolites in grapes. In fact, the response of the berry to abiotic stress induces the accumulation of secondary metabolites, as a defense mechanism against cell damages (Cramer et al., 2011), which contributes to improve the color, taste, and aroma of fresh and dried grapes and therefore drives an enhancement of grape quality.

To guarantee good quality issues in postharvest grapes, an innovative postharvest technology based on the treatment of grapes with ozone was developed for its effectiveness in decay control (Laureano et al., 2016) since it is an efficient and safe bactericidal and fungicidal agent (Botondi et al., 2015; Guzzon et al., 2018). Thereby, postharvest grape exposure to ozone has made it possible to reduce significantly the use of sulfur dioxide in winemaking (Mencarelli et al., 2011; Bellincontro et al., 2017). Despite these advantages, the influence of postharvest ozone treatments on stress-response secondary metabolites has scarcely been studied in grapes. Most of the studies have reported that ozone can stimulate berry chemical defense responses by synthetizing phenolic compounds (Carbone and Mencarelli, 2015; Bellincontro et al., 2017), mainly resveratrol (Artés-Hernández et al., 2003). Nevertheless, the elicitor effect of ozone on phenolic compounds is strongly dependent on the variety and on the duration and environmental conditions of the treatment (Botondi et al., 2015; Paissoni et al., 2017). Recently, Río Segade et al. (2017) have also observed for the first time that continuous ozone treatment during postharvest dehydration increases the contents of total volatile organic compounds (VOCs).

Volatile organic compounds have a significant impact on the organoleptic properties of grapes and wines. Postharvest dehydration of grapes is a widespread technique used for the production of fortified, reinforced, and sweet wines during which the concentration and/or synthesis of VOCs occur (Bellincontro et al., 2004; Costantini et al., 2006; Moreno et al., 2008). During grape dehydration, the berries sustain water loss and metabolic responses to water stress happen as a consequence of changes in gene expression pattern (Rizzini et al., 2009). However, gene expression and berry metabolism can be modulated during grape postharvest dehydration through the control of water loss rate (Rizzini et al., 2009). Different dehydration rates may activate differently the physiological response by modulating the activity of alcohol dehydrogenase (ADH) and lipoxygenase (LOX) enzymes, and the expression of *Vv*ADHs and carotenoid cleavage dioxygenase 1 (CCD1) genes (Chkaiban et al., 2007; Cirilli et al., 2012).

Degradation and transformation reactions of VOCs may also occur during grape postharvest dehydration (Bellincontro et al., 2004; Noguerol-Pato et al., 2013), particularly in aromatic varieties. In Moscato d’Alessandria grapes, a decrease of linalool, nerol, and geraniol contents was observed in the free fraction when the dehydration process progressed. On the contrary, bound terpenes showed good resistance to degradation reactions (Squadrito et al., 2009). Nevertheless, grape variety, ripeness grade and dehydration rate can also strongly influence the impact of postharvest dehydration on the volatile composition (Urcan et al., 2017). When postharvest dehydration was combined with a long-term ozone treatment, linalool, geraniol, and nerol, which are the major aromatic markers of Moscato bianco grapes, also increased as a consequence of important transcriptional changes occurring via methylerythritol phosphate (MEP) pathway that induced monoterpenes biosynthesis (Río Segade et al., 2017).

Taking into account that ozone is a powerful sanitizing agent and long-term ozone exposure during postharvest dehydration affects positively the contents of VOCs in aromatic white grapes, this research aimed to study for the first time the grape VOCs response to postharvest short-term ozone treatment. The effect of ozone concentration and exposure time was evaluated in both fresh and partially dehydrated (until reaching a weight loss of 20%) grapes just after treatment. This study was performed to better understand the changes produced by short-term ozone exposure in free and glycosylated VOCs at both postharvest grape status. The combined effect of grape ozone exposure and partial dehydration was also assessed. The study was carried out on *Vitis vinifera* L. cv. Moscato bianco due to the importance of preserving terpenes in aromatic white winegrapes to produce high quality dry wines.

## Materials and Methods

### Grape Sampling, Ozone Treatment, and Dehydration

Whole bunches of Moscato bianco (*V. vinifera* L.) grapes were harvested in 2015 when 27.0 ± 0.2° Brix were reached in the different sampling zones of a commercial vineyard located in the Piedmont wine region (Asti province, North-West Italy). Healthy whole bunches were divided in smaller clusters of 4–5 berries, which were randomly distributed in nine plastic boxes (30 cm × 20 cm, about 2 kg of grape berries per box). These boxes are perforated and the small clusters were arranged in a single layer for correct aeration. The first batch was exposed for 48 h to atmospheric air (control-F). Four batches were short-term treated with a normal dose of ozone (30 μL/L; Río Segade et al., 2017); the first two batches were ozone-exposed for 24 h and then air-exposed for 24 h (DN-24), while the second two batches were kept for 48 h under ozone-enriched air (DN-48). Other four batches were short-term treated with a high dose of ozone (60 μL/L); again the first two batches were ozone-exposed for 24 h and then air-exposed for 24 h (DH-24), while the second two batches were kept for 48 h under ozone-enriched air (DH-48). During the continuous ozone treatment into thermohygrometrically controlled chambers, an ozone generator (C32-AG, Industrie De Nora S.p.A, Milan, Italy) with a nominal production capacity of 32 g O_3_/h was used. The ozone generator output was continuously controlled with a BMT 964 UV-photometric ozone analyzer (BMT Messtechnik GmbH, DE) by monitoring the ozone-enriched air (120 m^3^/h flow) that is recirculated from the chamber.

One batch of grape berries for each one of the four ozone treatments was used for fresh grape analysis (DN-24-F, DN-48-F, DH-24-F, and DH-48-F). The first batch of control grape berries (not-treated with ozone) and the other batch for each of the four ozone treatments, all of about 2 kg, were withered into the thermohygrometrically controlled chambers until reaching a weight loss of 20% in 13 days. These samples were used for partially dehydrated grape analysis (control-D, DN-24-D, DN-48-D, DH-24-D, and DH-48-D).

The air exposure, ozone treatment and withering process were performed at 20 ± 2°C and 60 ± 5% relative humidity (RH) (Ossola et al., 2017; Río Segade et al., 2017). The environmental conditions were continuously monitored and recorded using a data logger (HOBO H8 RH/Temp, Onset Computer Corporation, Bourne, MA, United States).

### Determination of Standard Chemical Parameters

For each sample (fresh and partially dehydrated grapes) and for each of the five treatments (control, DN-24, DN-48, DH-24, and DH-48), three replicates of about 100 berries were randomly selected. For each replicate, the grape juice was obtained by manual crushing and centrifugation (7000 × *g*, 15 min). A high-performance liquid chromatography (HPLC) system (Agilent Technologies, Santa Clara, CA, United States) was used to determine tartaric acid, malic acid, citric acid, and glycerol (g/L) with a diode array detector (DAD) set to 210 nm, while reducing sugars (glucose and fructose, g/L) required a refractive index detector (Rolle et al., 2012).

### Extraction and Determination of Free and Glycosylated Volatile Compounds

For each sample and treatment, three replicates of about 100 g of grape berries were randomly selected for the determination of VOCs according to the method reported by Rolle et al. (2015). For each replicate, the berries were crushed for 1 min under a nitrogen atmosphere using a laboratory blender (Waring Laboratory, Torrington, CT, United States) and then centrifuged at 7000 × *g* for 15 min at 4°C. A 5-mL aliquot of the grape juice obtained was diluted with 5 mL of deionized water, adjusted to pH 5, and introduced into a 20-mL glass headspace sampling vial containing 2 g of sodium chloride. Then, 200 μL of internal standard consisting of a 1.55 mg/L 1-heptanol solution in 10% *v*/*v* ethanol were added for the free fraction analysis.

For the extraction of glycosylated VOCs, the method proposed by Wang et al. (2011) was used with some modifications. Briefly, 10 mL of the grape juice were loaded onto a 1-g Sep-Pak C18 cartridge (Waters Corporation, Milford, MA, United States), previously activated with methanol and washed with deionized water. The free fraction was eluted with 10 mL of dichloromethane and discarded. Once the cartridge was washed with 20 mL of deionized water, the glycosylated fraction was recovered with 10 mL of methanol and evaporated to dryness using a vacuum rotavapor (Buchi R-210, Switzerland) at 35°C. The residue was then dissolved in 5 mL of 0.2 M citrate-phosphate buffer at pH 5. The enzymatic hydrolysis was performed with 50 mg of an AR-2000 commercial preparation with glycosidase side activity (DSM Oenology, Netherlands) through incubation at 40°C for 21 h. This extract was diluted with an equal volume of citrate-phosphate buffer previously described and introduced into a 20-mL glass headspace sampling vial containing 2 g of sodium chloride. Then, 200 μL of internal standard (1.55 mg/L 1-heptanol solution in 10% *v*/*v* ethanol) were added for the glycosylated fraction analysis.

The VOCs determination by headspace solid-phase microextraction (HS-SPME) coupled with gas chromatography–mass spectrometry (GC–MS) required the use of a 50/30 μm divinylbenzene-carboxen-polydimethylsiloxane (DVB/CAR/PDMS) fiber (Supelco, Bellefonte, PA, United States), which was conditioned following the manufacturer’s recommendations. This fiber was exposed to the headspace of the capped vial for 20 min at 40°C (Saìnchez-Palomo et al., 2005) and the thermal desorption of analytes was performed at 250°C for 5 min for the splitless injection.

An Agilent 7890 C gas chromatograph (Santa Clara, CA, United States) coupled to an Agilent 5975 mass selective detector was used for VOCs identification and quantification following the instrumental conditions described by Saìnchez-Palomo et al. (2005) and modified by Rolle et al. (2015). A DB-WAXETR capillary column (30 m × 0.25 mm, 0.25 μm; J&W Scientific Inc., Folsom, CA, United States) was used with a temperature gradient from 40°C for 5 min, increasing at a rate of 2°C/min up to 200°C for 10 min and 5°C/min up to 220°C, and holding at 220°C for 5 min. The carrier gas was helium at a flow-rate of 1 mL/min. The mass acquisition range was 35–350 *m/z*. VOCs were determined (μg/L) using pure standards, purchased from Sigma-Aldrich (Milan, Italy) when available, and/or the NIST database^1^. Standard solutions were prepared in 10% *v*/*v* ethanol.

### Statistical Analysis

Regarding the experimental plan used in the study, while the design comprised the evaluation of the variability induced by ozone treatments on three different batches of grape berries for each treatment, the treatments themselves were conducted each in a single storage room. Therefore, although the experiment was carried out with a rigorous control of the operative conditions, the treatment replicates cannot be considered real independent statistical replicates. Due to this factor, for all tests the significance was tested at a higher-than-usual confidence level (*p* < 0.01). All data were statistically treated using the XLStat-Pro software from Addinsoft (Paris, France). Two-way analysis of variance (ANOVA) was applied, being ozone dose and exposure time the two main factors, and the HSD Tukey’s test for *p* < 0.01 was used to analyze significant differences among treatments for fresh and withered samples. Principal component analysis (PCA) was used to discriminate samples according to the determined free and glycosylated VOCs.

## Results

### Ozone Effect on Standard Chemical Parameters of Fresh and Withered Grapes

Table 1 shows the changes produced in the standard chemical parameters of fresh and withered (20% weight loss) grapes for Moscato bianco when ozone-enriched air was applied at different doses (DN and DH) and different short-term exposure times (24 and 48 h). In fresh grapes, no ozone treatment affected significantly the content of organic acids (tartaric acid, malic acid, and citric acid), reducing sugars (glucose and fructose), and glycerol with respect to control samples. However, high dose (DH) induced a slight increase of tartaric acid, glucose, and fructose contents versus normal dose (DN), although the dose effect was significant (*p* < 0.01) only for tartaric acid. Furthermore, the interaction between treatment dose and time (D^∗^T) was not significant for all parameters.

**Table 1:** Standard chemical composition (g/L) of fresh and withered Moscato bianco grapes after treatment with ozone at different exposure doses and times.

	Citric acid	Tartaric acid	Malic acid	Glucose	Fructose	Glycerol
**Fresh grapes**						
Control	0.16	6.80	1.44	134	142	0.00
DN-24	0.19	6.44	1.65	132	139	0.00
DN-48	0.16	6.39	1.71	134	142	0.00
DH-24	0.17	7.30	1.39	146	155	0.00
DH-48	0.18	7.58	1.53	159	169	0.08
Sig.	ns	ns	ns	ns	ns	ns
D	ns	^∗∗^	ns	ns	ns	ns
T	ns	ns	ns	ns	ns	ns
D^∗^T	ns	ns	ns	ns	ns	ns
**Withered grapes**						
Control	0.32a	7.73a	1.35	175	188	1.48a
DN-24	0.34a	6.15b	1.34	182	196	1.71a
DN-48	0.34a	6.19b	1.72	173	185	1.27ab
DH-24	0.22b	5.76b	1.44	175	188	0.97b
DH-48	0.25ab	6.27b	1.41	178	190	0.81b
Sig.	^∗∗^	^∗∗^	ns	ns	ns	^∗∗^
D	^∗∗^	ns	ns	ns	ns	^∗∗^
T	ns	ns	ns	ns	ns	^∗∗^
D^∗^T	ns	ns	ns	ns	ns	^∗∗^
**Fresh *versus* Withered grapes**						
Control	^∗∗^	^∗∗^	ns	^∗∗^	^∗∗^	^∗∗^
DN-24	^∗∗^	ns	ns	^∗∗^	^∗∗^	^∗∗^
DN-48	ns	ns	ns	ns	ns	^∗∗^
DH-24	ns	^∗∗^	ns	^∗∗^	^∗∗^	^∗∗^
DH-48	ns	ns	ns	ns	ns	^∗∗^


*Values are expressed as average (*n* = 3). Different Latin letters indicate significant differences among treatments for fresh and withered grape samples according to the HSD Tukey’s test (*p* < 0.01). Asterisks denote significant differences among treatments for fresh and withered grape samples or between fresh and withered samples for each treatment according to ANOVA: ^∗∗^indicates significance at *p* < 0.01; ns indicates no significant difference. DN, normal dose (30 μL/L); DH, high dose (60 μL/L); 24 and 48, 24 and 48 h of exposure, respectively; D, dose; T, time; and D^∗^T, dose–time interaction.*

In withered grapes, significant effects of ozone were reported for citric acid, tartaric acid, and glycerol among some treatments. Regarding acids, ozone exposure caused a significant decrease of citric acid (in DH samples) and tartaric acid versus control samples, corresponding the greatest reduction of their contents to DH-24 ozone-treated samples. No significant differences (*p* > 0.01) were found for malic acid, glucose and fructose in treated samples with respect to the control sample. On the other hand, DH ozone-exposed samples showed a significant decrease in the glycerol content versus control, the dose effect being more significant than the time effect.

When fresh and withered grapes were compared for each treatment, it was possible to observe that the dehydration process caused in the control sample a significant increase of all parameters except for malic acid (Table 1). This latter parameter did not change significantly as an effect of withering for all the treatments tested, while glycerol increased for all treatments. Furthermore, a positive withering influence in sugars (glucose and fructose), counterbalanced by a lower tartaric acid content, was significantly evidenced in DH-24 treatment.

### Ozone Effect on Total VOCs of Fresh and Withered Grapes

**FIGURE 1:**
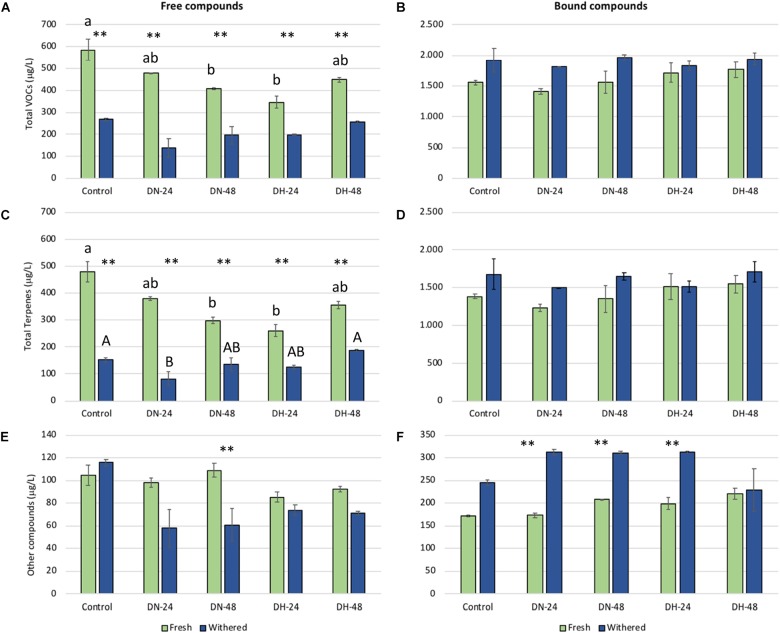
Total volatile composition (μg/L) in free and bound fractions of fresh and withered Moscato bianco grapes after treatment with ozone at different exposure doses and times. The figure shows the total content of volatiles **(A,B)**, terpenes **(C,D)**, and other non-terpene compounds **(E,F)** in the free **(A,C,E)** and glycosylated **(B,D,F)** fraction. Error bars correspond to standard deviations (*n* = 3). Values with different lower and capital Latin letters indicate significant differences among treatments for fresh and withered grape samples, respectively, according to the HSD Tukey’s test (*p* < 0.01). Asterisks (^∗∗^) denote a significant difference at *p* < 0.01 between fresh and withered samples for each treatment. DN, normal dose (30 μL/L); DH, high dose (60 μL/L); 24 and 48, 24 and 48 h of exposure, respectively.

The changes in the total content of volatiles, terpenes, and other compounds detected in fresh and withered Moscato bianco grapes by ozone exposure at different doses and times are shown in Figure 1. The free and bound fractions of VOCs in fresh and withered grapes presented different behavior due to the ozone effect. Regarding the free fraction, significant differences among treatments were observed for total VOCs and terpenes. In fresh grapes, total content of free VOCs and terpenes decreased in all ozone-treated samples versus control, with largest, significant (*p* < 0.01) reductions in DH-24 samples followed by DN-48. Furthermore, DH-24 ozone-treated samples also showed the lowest contents of the other non-terpene compounds even though the differences were not significant (*p* > 0.01) when compared to control. In withered grapes, total content of free terpenes decreased only for DN-24 with respect to control whereas all ozone treatments induced a not significant (*p* > 0.01) reduction of the other compounds. Furthermore, no significant differences (*p* > 0.01) among treatments were found in the bound fraction, independently on the ozone dose.

It is important to take into account that partial dehydration (20% weight loss) produced a significant decrease of total VOCs and terpene contents in the free fraction for control samples and all ozone-treated samples. However, the ozone treatment at the highest dose (DH) or for the longest time (48 h) reduced this decrease with respect to control up to by 12% for total VOCs and by 21% for total terpenes in DH-48 samples. The content of other free non-terpene compounds was not significantly affected by dehydration in control samples but a significant decrease was observed when ozone was applied at normal dose for 48 h (DN-48). Regarding the bound fraction, dehydration (20% weight loss) caused an increase of total VOCs, terpene, and other compound contents but this increase was significant only for other non-terpene compounds, for which DN-24, DN-48, and DH-24 ozone treatments intensified this increase by 15, 3, and 6%, respectively, with respect to control.

### Ozone Effect on Free Volatile Compounds of Fresh and Withered Grapes

Table 2 shows the content of individual free volatile compounds in fresh and withered Moscato bianco grapes when ozone was applied at different doses and exposure times. A total of 25 free volatile compounds were determined (17 terpenes, 6 alcohols, and 2 aldehydes). The dose and time of ozone exposure had different effects depending on the analyzed compound. A significant effect of the ozone dose was observed in fresh grapes for linalool, benzyl alcohol, and benzaldehyde contents whereas in withered grapes it was for (+)-4-carene, *cis*-furan linalool oxide, α-terpineol, and 2-phenylethanol. The exposure time affected significantly benzaldehyde contents in fresh grapes whereas it did for 4-terpineol, and 2-phenylethanol in withered grapes. The interaction D^∗^T was significant only for linalool and benzaldehyde in fresh and withered grapes, and 4-terpineol and benzaldehyde in withered grapes.

**Table 2:** Free volatile composition (μg/L) of fresh and withered Moscato bianco grapes after treatment with ozone at different exposure doses and times.

Free Compounds	Fresh grapes						Withered grapes						Sig. Fresh *versus* Withered				
					
	Control	DN-24	DN-48	DH-24	DH-48	Sig.	Control	DN-24	DN-48	DH-24	DH-48	Sig.	Control	DN-24	DN-48	DH-24	DH-48
D-Limonene	5.89	4.71	4.78	7.64	6.28	ns	14.40a	1.13b	1.27b	2.27b	2.82b	ns	ns	ns	ns	ns	ns
β-Phellandrene	3.14	3.39	1.40	3.79	3.56	ns	1.33	0.46	2.46	0.90	0.05	ns	ns	ns	ns	ns	ns
(+)-4-Carene	1.19	1.11	1.02	0.87	1.26	ns	0.71bc	0.18c	1.16b	1.11b	2.12a	^∗∗^	ns	^∗∗^	ns	ns	^∗∗^
*trans*-Rose oxide	7.06	6.59	6.09	5.70	6.70	ns	4.35	2.23	1.33	2.74	3.60	ns	ns	^∗∗^	^∗∗^	ns	ns
*cis*-Rose oxide	1.62	1.56	1.55	1.44	1.66	ns	0.94	0.41	0.55	0.69	0.83	ns	ns	^∗∗^	^∗∗^	^∗∗^	^∗∗^
*trans*-Furan linalool oxide	1.63	1.27	1.48	1.05	0.59	ns	0.61	0.24	0.38	0.29	0.69	ns	ns	ns	ns	ns	ns
*cis*-Furan linalool oxide	2.09	1.51	1.81	1.90	2.20	ns	0.97ab	0.51b	0.77ab	1.00ab	1.60a	^∗∗^	^∗∗^	^∗∗^	^∗∗^	ns	ns
Linalool	331.53a	243.80b	174.61bc	124.99c	215.42b	^∗∗^	50.22	41.96	49.16	55.37	72.08	ns	^∗∗^	^∗∗^	^∗∗^	^∗∗^	^∗∗^
Ho-trienol	0.98	0.85	0.99	0.60	0.81	ns	0.29	0.23	0.26	0.29	0.96	ns	ns	ns	ns	ns	ns
Geranial	0.59	0.90	0.83	0.23	0.80	ns	0.81	0.31	0.14	0.28	0.60	ns	ns	ns	ns	ns	ns
α-Terpineol	1.67	1.59	1.79	1.58	2.51	ns	1.01ab	0.43b	0.69ab	0.93ab	1.43a	^∗∗^	ns	^∗∗^	^∗∗^	ns	^∗∗^
Neral	4.22a	3.44ab	2.94b	2.89b	2.68b	^∗∗^	2.55	0.88	0.94	1.36	1.44	ns	^∗∗^	^∗∗^	^∗∗^	^∗∗^	ns
*trans*-Pyran linalool oxide	3.16	2.88	3.04	2.45	2.77	ns	1.64	0.83	1.25	1.75	2.21	ns	^∗∗^	^∗∗^	^∗∗^	ns	ns
Citronellol	6.75	6.74	6.18	6.32	6.47	ns	5.25	2.05	2.30	3.10	2.89	ns	ns	^∗∗^	^∗∗^	^∗∗^	^∗∗^
Nerol	39.50	35.00	33.08	36.77	36.68	ns	28.27a	12.74b	11.38b	11.86b	15.33b	^∗∗^	ns	^∗∗^	^∗∗^	^∗∗^	^∗∗^
Geraniol	69.74	64.27	56.97	62.46	65.41	ns	39.40	17.57	18.27	22.53	23.23	ns	^∗∗^	^∗∗^	^∗∗^	^∗∗^	^∗∗^
4-Terpineol	nd	nd	nd	0.13	0.48	ns	nd	nd	43.82a	19.39b	54.37a	^∗∗^	ns	ns	^∗∗^	^∗∗^	^∗∗^
Pentanol	0.57	1.17	1.69	1.44	0.80	ns	1.25	0.89	1.07	0.96	1.61	ns	ns	ns	ns	ns	ns
3-Methyl-2-buten-1-ol	0.90	0.74	0.96	0.63	0.41	ns	0.62	0.36	0.33	0.44	0.57	ns	ns	ns	ns	ns	ns
Hexanol	84.96	79.21	87.29	65.74	68.58	ns	85.07	47.39	38.63	58.56	54.28	ns	ns	ns	^∗∗^	ns	ns
2-Ethylhexanol	8.04	7.41	8.62	7.00	7.46	ns	5.74	3.85	4.16	4.29	6.09	ns	ns	ns	^∗∗^	ns	ns
Benzyl alcohol	1.99b	2.06ab	2.79ab	3.18a	3.10ab	^∗∗^	1.74	0.83	0.93	1.31	1.47	ns	ns	^∗∗^	^∗∗^	^∗∗^	^∗∗^
2-Phenylethanol	5.11a	4.16b	3.97b	3.78b	3.83b	^∗∗^	3.85a	1.38b	3.11ab	2.78ab	4.08a	^∗∗^	ns	^∗∗^	ns	^∗^	ns
Octanal	0.46	0.64	0.65	0.48	0.54	ns	0.39	0.54	0.55	0.62	0.49	ns	ns	ns	ns	ns	ns
Benzaldehyde	2.68b	2.65b	2.94b	3.23b	7.80a	^∗∗^	17.21a	2.56b	12.02ab	4.91b	2.64b	^∗∗^	^∗∗^	ns	^∗∗^	ns	ns


*Values are expressed as average (*n* = 3). Different Latin letters indicate significant differences among treatments for fresh and withered grape samples according to the HSD Tukey’s test (*p* < 0.01). Asterisks denote significant differences among treatments for fresh and withered grape samples or between fresh and withered samples for each treatment: ^∗∗^indicates significance at *p* < 0.01; ns indicates no significant difference. DN, normal dose (30 μL/L); DH, high dose (60 μL/L); 24 and 48, 24 and 48 h of exposure, respectively.*

Regarding fresh grapes, linalool, hexanol, geraniol, and nerol were the most abundant free volatile compounds in Moscato bianco grapes (Table 2). A significant effect due to ozone exposure was found for five compounds in the free fraction versus control. However, only benzyl alcohol, and benzaldehyde showed a significant increase of their content, particularly in DH-24 ozone-treated samples for the former and in DH-48 for the latter. All ozone treatments affected negatively free linalool contents. The other most abundant free terpenes in Moscato bianco grapes (nerol and geraniol) were not significantly affected by short-term ozone treatments in relation to control grapes. As occurred for linalool, ozone impacted negatively free neral and 2-phenylethanol contents independently on the ozone concentration and exposure time.

In withered grapes, a stronger effect of ozone was observed because seven free volatile compounds showed significant differences among treatments (Table 2). Ozone applied at DH-48 induced a significant increase of (+)-4-carene whereas it did at DH-24, DN-48, and DH-48 for 4-terpineol with respect to control. Most of free volatile compounds evidenced a similar content to control in the samples treated with the high ozone dose (DH), including linalool, geraniol, and hexanol. Nevertheless, it is important to take into account that a tendency to increase the content was evidenced for linalool, *cis*-furan linalool oxide, α-terpineol, and *trans*-pyran linalool oxide, although not significant, when ozone was applied at a high dose (DH-48). Only three free compounds in withered grapes, such as D-limonene, nerol, and benzaldehyde, reduced significantly their content in ozone-treated samples versus control independently on the dose and exposure time whereas hexanol and neral showed a non-significant tendency to decrease in short-term treatments at the normal dose (DN-24 and DN-48 samples).

Table 2 also shows the significance of the differences in free volatiles between fresh and withered grapes for each treatment with the aim to compare the effect of dehydration at 20% weight loss after subjecting the samples to different ozone treatments. In control samples, dehydration affected significantly six volatile compounds, increasing only the content of D-limonene and benzaldehyde. The content of some free terpenes, such as neral, geraniol, and linalool, decreased significantly in withered grapes with respect to fresh samples in control and in all ozone-treated samples. Indeed, a decrease of –85% was found for linalool in control samples but the DH treatment reduced this decrease ranging from –56 to –66%. Among the other free terpenes, ozone induced a significant increase of 4-terpineol contents for DN-48, DH-24, and DH-48 treatments for withered versus fresh samples. (+)-4-Carene also showed the same behavior in DH-48 ozone-treated samples. Furthermore, the ozone treatment at the high dose (DH) permitted to overturn partially the decrease observed in DN samples by dehydration effect for *trans*-rose oxide, *trans*-pyran linalool oxide, ho-trienol, hexanol, and 2-ethylhexanol and even in control samples for *trans*-pyran linalool oxide and ho-trienol. Other free compounds, such as benzyl alcohol, exhibited an opposite response because ozone caused the increase of the content in fresh but did not in withered grapes.

### Ozone Effect on Glycosylated Volatile Compounds of Fresh and Withered Grapes

Table 3 shows the glycosidically bound volatile composition of fresh and withered Moscato bianco grapes air-treated and short-term ozone-treated at different doses and exposure times. A total of 23 compounds were determined in the bound fraction (16 terpenes, 5 alcohols, and 2 aldehydes). A significantly higher (*p* < 0.01) effect of the ozone dose versus the time exposure was observed in withered grapes for ho-trienol and benzyl alcohol (Table 4). Instead, the effect of exposure time was more significant in fresh grapes for hexanol, and in withered grapes for nerol, and geraniol. Bound *cis*-rose oxide and ho-trienol exhibited significant D^∗^T interaction, and only in withered grapes (Table 4).

**Table 3:** Glycosidically bound volatile composition (μg/L) of fresh and withered Moscato bianco grapes after treatment with ozone at different exposure doses and times.

Bound compounds	Fresh grapes						Withered grapes						Sig. Fresh *versus* Withered				
					
	Control	DN-24	DN-48	DH-24	DH-48	Sig.	Control	DN-24	DN-48	DH-24	DH-48	Sig.	Control	DN-24	DN-48	DH-24	DH-48
D-Limonene	22.27	17.78	21.65	21.35	17.39	ns	17.82	19.72	32.05	20.07	22.18	ns	ns	ns	ns	ns	ns
β-Phellandrene	13.84	11.14	13.51	14.39	12.08	ns	11.54b	13.20ab	17.53a	12.54b	13.05ab	^∗∗^	ns	ns	ns	ns	ns
(+)-4-Carene	5.29	4.65	5.75	5.71	4.91	ns	5.79	5.12	7.90	5.15	6.06	ns	ns	ns	ns	ns	ns
*trans*-Rose oxide	9.26	13.19	18.07	12.33	19.25	ns	33.67a	16.86ab	20.36ab	19.54ab	13.58b	^∗∗^	^∗∗^	ns	ns	ns	ns
*cis*-Rose oxide	3.23	4.36	6.16	4.02	6.08	ns	10.16a	5.83b	6.69b	6.50b	4.12b	^∗∗^	^∗∗^	ns	ns	ns	ns
*tran*s-Furan linalool oxide	11.01	7.39	6.65	7.66	9.74	ns	16.96	9.47	9.66	10.18	7.39	ns	ns	ns	ns	ns	ns
*cis*-Furan linalool oxide	1.34	1.01	0.96	1.13	1.25	ns	2.50	1.15	1.27	1.28	1.01	ns	ns	ns	ns	ns	ns
Linalool	458.97	360.03	327.40	414.64	470.10	ns	574.83	518.29	456.08	499.20	482.21	ns	ns	ns	ns	ns	ns
Ho-trienol	3.42	2.55	2.84	3.31	3.33	ns	3.21a	3.64a	3.10a	3.01a	1.04b	^∗∗^	ns	ns	ns	ns	ns
Geranial	6.04	28.05	32.53	41.39	37.33	ns	15.44	28.39	34.52	30.29	33.80	ns	ns	ns	ns	ns	ns
α-Terpineol	8.93	6.47	6.77	11.39	18.09	ns	20.13	17.43	20.68	22.19	13.76	ns	ns	ns	ns	ns	ns
Neral	10.63	21.79	25.72	33.20	33.67	ns	19.15	24.87	33.15	25.04	24.02	ns	ns	ns	ns	ns	ns
*trans*-Pyranlinalool oxide	2.58	1.85	1.72	2.44	2.52	ns	4.40	2.85	3.05	2.59	2.19	ns	ns	ns	ns	ns	ns
Citronellol	24.52	24.40	38.01	32.48	34.43	ns	36.50	33.11	38.40	32.26	38.66	ns	ns	ns	ns	ns	ns
Nerol	457.53	405.73	482.10	504.45	493.28	ns	481.07	439.12	533.46	458.87	575.84	ns	ns	ns	ns	ns	ns
Geraniol	342.66	326.00	364.00	404.28	384.38	ns	424.37	365.90	432.87	366.71	472.42	ns	ns	ns	ns	ns	ns
Pentanol	6.35	5.79	7.33	6.18	8.11	ns	6.18	10.26	10.29	11.07	7.30	ns	ns	ns	ns	ns	ns
3-Methyl-2-buten-1-ol	7.62	6.99	6.71	5.93	6.74	ns	4.11	7.56	7.75	6.71	1.73	ns	ns	ns	ns	ns	ns
Hexanol	123.14b	127.20ab	159.44ab	145.14ab	165.34a	^∗∗^	202.09	248.70	237.69	256.33	195.52	ns	ns	^∗∗^	ns	^∗∗^	ns
Benzyl alcohol	10.83	10.80	10.67	12.82	13.64	ns	8.17ab	14.20ab	16.32a	11.16ab	3.19b	^∗∗^	ns	ns	ns	ns	^∗∗^
2-Phenylethanol	18.15	17.61	18.77	23.39	22.80	ns	21.66	25.65	31.26	23.05	17.47	ns	ns	ns	ns	ns	ns
Octanal	1.50	1.48	1.85	2.15	0.95	ns	0.58	2.32	1.47	1.14	2.04	ns	ns	ns	ns	ns	ns
Benzaldehyde	3.98	3.43	3.24	3.78	3.34	ns	2.60	3.87	6.35	3.45	1.69	ns	ns	ns	ns	ns	ns


*Values are expressed as average (*n* = 3). Different Latin letters indicate significant differences among treatments for fresh and withered grape samples according to the HSD Tukey’s test (*p* < 0.01). Asterisks denote significant differences among treatments for fresh and withered grape samples or between fresh and withered samples for each treatment: ^∗∗^indicates significance at *p* < 0.01; ns indicates no significant difference. DN, normal dose (30 μL/L); DH, high dose (60 μL/L); 24 and 48, 24 and 48 h of exposure, respectively.*

**Table 4:** Two-way ANOVA results on free and glycosidically bound volatile composition (μg/L) of fresh and withered Moscato bianco grapes after treatment with ozone at different exposure doses and times.

	Free volatile compounds						Glycosidically bound compounds					
		
	Fresh grapes			Withered grapes			Fresh grapes			Withered grapes		
				
	D	T	D^∗^T	D	T	D^∗^T	D	T	D^∗^T	D	T	D^∗^T
D-Limonene	ns	ns	ns	ns	ns	ns	ns	ns	ns	ns	ns	ns
β-Phellandrene	ns	ns	ns	ns	ns	ns	ns	ns	ns	^∗∗^	^∗∗^	ns
(+)-4-Carene	ns	ns	ns	^∗∗^	^∗∗^	ns	ns	ns	ns	ns	ns	ns
*trans*-Rose oxide	ns	ns	ns	ns	ns	ns	ns	ns	ns	ns	ns	ns
*cis-*Rose oxide	ns	ns	ns	ns	ns	ns	ns	ns	ns	ns	ns	^∗∗^
*trans*-Furan linalool oxide	ns	ns	ns	ns	ns	ns	ns	ns	ns	ns	ns	ns
*cis*-Furan linalool oxide	ns	ns	ns	ns	ns	ns	ns	ns	ns	ns	ns	ns
Linalool	ns	ns	^∗∗^	^∗∗^	ns	ns	ns	ns	ns	ns	ns	ns
Ho-trienol	ns	ns	ns	ns	ns	ns	ns	ns	ns	^∗∗^	^∗∗^	^∗∗^
Geranial	ns	ns	ns	ns	ns	ns	ns	ns	ns	ns	ns	ns
α-Terpineol	ns	ns	ns	ns	ns	ns	ns	ns	ns	ns	ns	ns
Neral	ns	ns	ns	ns	ns	ns	ns	ns	ns	ns	ns	ns
*trans*-Pyran linalool oxide	ns	ns	ns	ns	ns	ns	ns	ns	ns	ns	ns	ns
Citronellol	ns	ns	ns	ns	ns	ns	ns	ns	ns	ns	ns	ns
Nerol	ns	ns	ns	ns	ns	ns	ns	ns	ns	ns	^∗∗^	ns
Geraniol	ns	ns	ns	ns	ns	ns	ns	ns	ns	ns	^∗∗^	ns
4-Terpineol	ns	ns	ns	ns	^∗∗^	^∗∗^	*nd*	*nd*	*nd*	*nd*	*nd*	*nd*
Pentanol	ns	ns	ns	ns	ns	ns	ns	ns	ns	ns	ns	ns
3-Methyl-2-buten-1-ol	ns	ns	ns	ns	ns	ns	ns	ns	ns	ns	ns	ns
Hexanol	ns	ns	ns	ns	ns	ns	ns	^∗∗^	ns	ns	ns	ns
2-Ethylhexanol	ns	ns	ns	ns	ns	ns	*nd*	*nd*	*nd*	*nd*	*nd*	*nd*
Benzyl alcohol	^∗∗^	ns	ns	ns	ns	ns	ns	ns	ns	^∗∗^	ns	ns
2-Phenylethanol	ns	ns	ns	ns	^∗∗^	ns	ns	ns	ns	ns	ns	ns
Octanal	ns	ns	ns	ns	ns	ns	ns	ns	ns	ns	ns	ns
Benzaldehyde	^∗∗^	^∗∗^	^∗∗^	ns	ns	^∗∗^	ns	ns	ns	ns	ns	ns


*Asterisks denote significant differences among treatments for fresh and withered grape samples according to two-way ANOVA: ^∗∗^indicates significance at *p* < 0.01; ns indicates no significant difference. *nd* indicates no detected. D, dose; T, time; and D^∗^T, dose–time interaction.*

Regarding fresh grapes (Table 3), only bound hexanol, which is the most abundant alcohol in Moscato bianco grapes, was influenced by ozone treatment, increasing significantly (*p* < 0.01) its content with respect to control when ozone at high dose was applied during 48 h. Instead, significant differences were not observed among treatments for the most abundant bound terpenes in Moscato bianco grapes, such as linalool, geraniol, and nerol. However, a slight trend to increase the content of these latter compounds was observed in fresh grapes, although not significant versus control, when ozone was applied at the high dose (DH) as also occurred for other bound terpenes.

In withered grapes, ozone treatments exhibited influence on five bound volatile compounds (four terpenes and one alcohol compound) with respect to control sample (Table 3). The most abundant bound volatile compounds, namely linalool, nerol, and geraniol, were not affected significantly by ozone treatments. However, a slight but not significant trend to increase nerol and geraniol contents was observed when DN-48 and DH-48 ozone treatments were applied. By contrast, the contents of bound rose oxides (*cis-* and *trans*-isomers) were negatively influenced by ozone treatments in withered grapes, corresponding the lowest values to DH-48 sample. The content of bound ho-trienol also decreased significantly for DH-48 ozone-treated grapes with respect to the control.

Table 3 also shows the significant differences in bound volatile compounds between fresh and withered grapes for each treatment, which affected a smaller number of compounds compared to free volatile composition. In control samples, only two compounds (*cis-*rose oxide and *trans-*rose oxide) showed significant (*p* < 0.01) differences, increasing their content with dehydration. Furthermore, ozone affected fresh and withered grapes in a quite similar way because only two bound volatile compounds exhibited different behavior between the two grape status for ozone-treated samples. Among these compounds, only hexanol also showed an increased content in withered versus fresh grapes for DN-24 and DH-24 ozone-treatments. However, the content of benzyl alcohol decreased in withered versus fresh grapes when DH-48 ozone treatment was applied.

### Differentiation of Ozone-Treated Grapes According to Free and Glycosylated Volatile Composition

**FIGURE 2:**
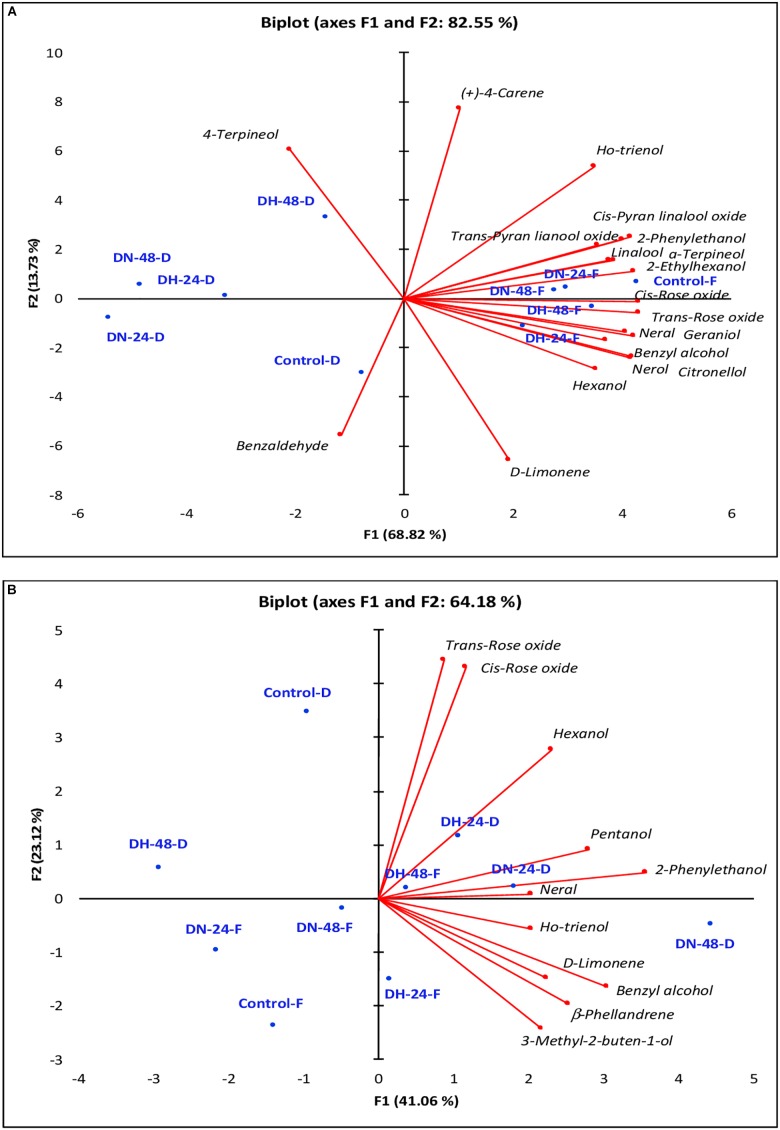
Principal component analysis (PCA) applied to the volatile composition in free **(A)** and bound **(B)** fractions of fresh and withered Moscato bianco grapes after treatment with ozone at different exposure doses and times. DN, normal dose (30 μL/L); DH, high dose (60 μL/L); 24 and 48, 24 and 48 h of exposure, respectively; F, fresh; D, withered.

To better visualize the differentiation among ozone treatments in fresh and withered Moscato bianco grapes, two PCAs were performed on the free and glycosylated fractions of VOCs with significantly different contents among samples (Figure 2).

Figure 2A shows the PCA performed on free volatile compounds. The first two principal components (PC1 and PC2) accounted for 82.55% of the total variance (68.82 and 13.73%, respectively). Two well-defined groups were differentiated on the basis of the grape status (fresh and withered). The first group was formed by fresh grapes (control and ozone-treated), which were placed in the positive side of PC1. The second group included the control and all ozone-treated withered grapes, which were located in the negative side of PC1. Nevertheless, control and DH-48 withered samples were also associated with the negative and positive side of PC2, respectively. Most of free volatile compounds were highly and positively correlated to the PC1, except for (+)-4-carene with an important positive contribution of PC2 and D-limonene with a negative contribution of PC2. Two compounds (4-terpineol and benzaldehyde) were negatively correlated to PC1. However, 4-terpineol also contributed positively to PC2 whereas benzaldehyde did negatively. This confirmed the decrease of most free compounds with dehydration at 20% weight loss but DH-48 withered sample was particularly rich in 4-terpineol. Therefore, short-term ozone treatment promotes the synthesis of 4-terpineol for exposure times of 48 h at high doses (60 μL/L).

Figure 2B shows the PCA conducted on glycosylated volatile compounds. The first two principal components (PC1 and PC2) accounted for 64.18% of the total variance (41.06 and 23.12%, respectively). In bound compounds, mainly the grape status (fresh and withered) also contributed to the differentiation among samples. Most of fresh grape samples (ozone-treated and control) were associated with the highest negative values of PC1 and PC2. On the other hand, withered grape samples were distributed into two groups: one group located in the positive side of PC1 (DH-24-D, DN-24-D, and DN-48-D) and characterized by high contents of most bound compounds, and a second group formed by control-D and DH-48-D and sited in the positive side of PC2 and the negative side of PC1, respectively. This different behavior of DH-48-D sample could be due to the lower contents of rose oxide isomers, ho-trienol, 3-methyl-2-buten-1-ol, and benzyl alcohol, as above mentioned.

Therefore, untreated and ozone-treated fresh grapes were characterized by high contents of most of free volatile compounds, mainly terpenes. However, high contents of bound compounds were associated with withered grapes treated with ozone at different doses and exposure times.

## Discussion

This study describes the changes caused by short-term ozone treatments on metabolites of fresh and withered Moscato bianco grapes. In agreement with our results for fresh grapes, other studies performed on Petit Verdot red grapes exposed for 12 h to a maximum flow of 20 g/h with 6% *w*/*w* ozone at 4°C reported that total sugar contents and titratable acidity values were not influenced by the ozone treatment (Bellincontro et al., 2017). Also in fresh Pignola red grapes shock ozone-treated for 18 h with a continuous flow of 1.5 g/h ozone at 10°C, differences were not evidenced in both total soluble solids and titratable acidity with respect to control (Botondi et al., 2015).

Nevertheless, in the present work, a non-significant (*p* > 0.01) tendency to increase reducing sugar contents, both glucose and fructose, was observed in fresh and withered Moscato bianco grapes in response to short-term ozone treatments with respect to control samples, particularly fructose in withered grapes. This non-significant increasing trend was higher for DH-48 fresh grapes (+18–19%) than for DN-24 withered grapes (+4%). In a previous study, a significantly higher content of total soluble solids was found in short-term ozone-exposed Grechetto white grapes using a continuous flow of 1.5 g/h ozone-enriched air for 12 h at 10°C (Carbone and Mencarelli, 2015). Furthermore, a significant increase of glucose and fructose contents was observed in withered Moscato bianco grapes at 20% weight loss when they were continuously exposed to an ozone dose of 30 μL/L at 20°C (Río Segade et al., 2017). In contrast, other authors (Botondi et al., 2015) reported that the total sugar content was not influenced by the ozone treatment in Pignola red grapes withered at 20% weight loss after shock treatment for 18 h with a continuous flow of 1.5 g/h ozone at 10°C or even during long-term exposure using the shock treatment followed by dehydration in normal atmosphere applying 0.5 g/h ozone for 4 h each day.

Malic acid is more susceptible to abiotic stress than tartaric and citric acids as a consequence of its involvement in gluconeogenesis, respiration, and secondary compounds biosynthesis (Sweetman et al., 2009). However, we have observed that the shock treatment for 24 h with a high ozone dose (60 μL/L) caused the reduction of tartaric and citric acids in withered samples whereas that of malic acid could have been partially overturned by the concentration effect due to dehydration, as reported by Río Segade et al. (2017) for weight losses higher than 15% in Moscato bianco grapes under long-term ozone treatment. According to the results obtained in this study, the short-term ozone treatment may affect the grape acid metabolism during subsequent dehydration just as the double stress response to dehydration and ozone could influence the acid metabolism during long-term treatment. In fact, this double stress response was hypothesized by Botondi et al. (2015) who also observed a different trend of titratable acidity values depending on the ozone treatment (shock or long-term) in Pignola red grapes partially dehydrated at 20% weight loss.

Plants respond to biotic and abiotic stresses through a chemical defense response, synthesizing specific metabolites such as phenolic and volatile compounds (Carbone and Mencarelli, 2015). Abiotic stress produced by postharvest dehydration changed markedly the volatile composition of Malvasia, Trebbiano, and Sangiovese grapes (Bellincontro et al., 2004) as well as of Aleatico grapes (Cirilli et al., 2012). In agreement with our results, in Moscato d’Alessandria white aromatic grapes, decreased terpene contents were found in the free fraction when the dehydration process progressed whereas bound terpenes were resistant to degradation (Squadrito et al., 2009). In Malvasia moscata white grapes, total glycosylated terpene contents increased significantly after dehydration up to 20% weight loss, exceeding the concentration effect by water evaporation (Urcan et al., 2017). Furthermore, in Moscato bianco grapes, a decrease of total free terpenes was observed in the last ripening stages (Brix > 19.3°) but the content of total bound terpenes increased progressively during ripening (Torchio et al., 2016). Therefore, in the present work, the differences reported between fresh and withered samples for total terpenes in control (air-treated grapes) agreed with what happened in the last ripening stages of Moscato bianco grapes.

Ozone also is an abiotic stressor that can induce changes in the aromatic profile of postharvest grapes. The results obtained in this work showed that total contents of free VOCs decreased by a high-dose short-term ozone treatment in fresh grapes with respect to control samples, mainly due to terpenes, but they remained relatively stable in withered grapes. In fresh fruits, the oxidation of volatile compounds was hypothesized by Nadas et al. (2003) as responsible for the loss of sensory evaluated aroma when storage was performed in cold ozone-enriched atmosphere. Ozone is a powerful oxidant acting through both direct reactions with the target organic compound by attaching itself to the double bond and indirect reactions by the formation of intermediate hydroxyl radicals. The oxidation of aromatic compounds can occur by this last mechanism with simultaneous degradation and formation of compounds (Cullen et al., 2009). Contrarily, Pérez et al. (1999) evidenced that enzymes involved in the biosynthesis of volatile compounds were not influenced by the presence of ozone and therefore they supposed that the physical and chemical changes in the fruit surface layers could reduce the emission of these compounds in ozone-treated fruits. Nevertheless, the effects of ozone on the physiology and quality of grapes vary according to the variety, ripening stage, environmental conditions, ozone concentration, exposure time, and application type (Artés-Hernández et al., 2007; Gabler et al., 2010; Feliziani et al., 2014; Botondi et al., 2015; Paissoni et al., 2017). In cold storage of table grapes, differences in flavor were not detectable when they were treated with ozone at 0.300 μL/L compared to those stored in air (Feliziani et al., 2014) whereas adverse effects were found at 2 μL/L ozone applied in continuous or intermittent mode (Cayuela et al., 2009).

Recently, Río Segade et al. (2017) published the first study on the ozone effect on the volatile composition of grapes during long-term and continuous exposure. They highlighted important information on the impact of ozone on the synthesis of VOCs during postharvest grape dehydration under controlled thermohygrometric conditions. These authors suggested that long-term ozone treatment promotes the synthesis of many volatile compounds that can contribute positively to grape and wine aromatic quality, particularly terpenes in aromatic varieties, through the up-regulation of several genes involved. As above mentioned, in the present study, short-term ozone treatments did not affect substantially total content of free VOCs in withered grapes. The lower ozone dose (DN) and the shorter exposure time (24 h) caused a significant decrease of total free terpenes and VOCs in withered grapes but when using higher doses and/or longer treatment times the differences were shortened. After an initial reduction, the dose and time of short-term ozone exposure seem to induce the formation of grape free terpenes in this study, as it happened in long-term ozone treatment during the dehydration process (Río Segade et al., 2017).

In the present study, total bound VOCs resulted to be less sensitive to the ozone treatments, their contents remaining quite stable after the short-term ozone exposure with respect to control in both fresh and withered grapes. Only a slight, non-significant (*p* > 0.01) tendency to increase total bound terpenes and volatile compounds was observed in fresh grapes when a high ozone dose (DH) was applied whereas this increase was reduced in withered grapes.

The VOCs emission from plants by multiple stress factors was reviewed by Holopainen and Gershenzon (2010). Additive effects of abiotic stresses acting through the same biochemical mechanism could occur. Nevertheless, the plant response to multiple stresses may be prioritized against those most severe or greatest threatening to survival, or those for which the most resources are available for mitigation (Holopainen and Gershenzon, 2010). Botondi et al. (2015) hypothesized the existence of cumulative oxidative stress (ozone and water loss), which could be the cause of the different pattern observed in our study for VOCs in fresh and partially dehydrated grapes after short-term ozone treatment. In fact, the combined effect of ozone and dehydration on volatile compounds was studied by De Sanctis et al. (2015), who applied ozone during grape dehydration observing that this combination induced the decrease of free volatiles in Sauvignon blanc grapes versus untreated ones, whereas glycosylated compounds increased.

Terpenes are responsible for floral and fruity aroma and are the dominant aromatic markers of Moscato bianco grapes. The stimulation of the terpene biosynthesis by oxidative stress has been reported by several authors in fruits and plants (Loreto et al., 2004; Beaulieu, 2007; Kegge and Pierik, 2010). The emission of these compounds can be stimulated in response to a high dose of ozone whereas it is often reduced when the plants are exposed to low doses. Studies revealed that terpene biosynthesis is induced when the ozone dose exceeds a threshold that marks the cellular damage (Loreto and Schnitzler, 2010). In fact, Holopainen and Gershenzon (2010) reported that ozone can cause tissue damage by reacting reactive oxygen species with lipids, proteins or other cellular components, which increases the VOC emission as stress response.

Some individual volatiles, mainly isoprene, monoterpenes, and sesquiterpenes, have specific roles in various stress responses (Holopainen and Gershenzon, 2010). Among terpenes, in the present study, all short-term ozone treatments negatively affected free linalool contents in fresh grapes, although those treatments with the normal ozone concentration at the shorter exposure time (DN-24) and with the high ozone concentration at the longer exposure time (DH-48) showed the smaller losses for this compound. It could be hypothesized that the less stressful ozone treatment caused the smaller degradation of free linalool. However, a higher stress response against the strong ozone treatment induced the synthesis of this compound, as also occurred in withered grapes. The dehydration process significantly decreased the contents of free linalool, nerol (except for control), and geraniol, which are the most important terpenes in Muscat varieties. However, the short-term ozone treatment with the high dose (DH) reduced the loss of free linalool by water stress. Therefore, ozone induced a beneficial stress response in free linalool content. Bound linalool, nerol, and geraniol were not affected by ozone treatment in fresh and withered grapes.

In our study, the content of other free and bound terpenes (free (+)-4-carene, free 4-terpineol, and bound β-phellandrene in withered grapes) increased when ozone was applied at short-term versus control. It is important to take into account that ozone seems to elicit the synthesis of free (+)-4-carene (DH-48 samples) and free 4-terpineol (DN-48 and DH-48 samples) in withered grapes.

The isoprene emission enhanced by ozone application is controlled at a transcriptional level by the greatest isoprene synthase messenger RNA (ISPS mRNA) expression (Fares et al., 2006), which up-regulates the ISPS protein levels. When a moderate or low dose of ozone is applied, the induction of the terpene pathway as stress response to ozone is absent and the amount of ISPS protein may be reduced (Calfapietra et al., 2007). Other studies support the hypothesis that the terpene metabolism is modulated according to the treatment intensity. Low stress promotes the synthesis of enzymes involved in an acclimation response whereas high stress induces the MEP pathway (Gil et al., 2012). Río Segade et al. (2017) demonstrated that postharvest dehydration combined with long-term ozone exposure induces the biosynthesis of monoterpenes via the MEP pathway in Moscato bianco grapes. In the present work, the slightly non-significantly (*p* > 0.01) higher contents of free linalool and bound geraniol, as well as the significantly higher amounts of bound β-phellandrene, in grapes short-term ozone-treated for 48 h and subsequently withered at 20% weight loss quite agreed with the higher transcriptional levels of linalool, geraniol, and phellandrene synthases (*Vv*LYN, *Vv*GERAN, and *Vv*PHELL, respectively) found at the end of the dehydration process (between 15 and 30% weight loss) under long-term ozone exposure (Río Segade et al., 2017).

In addition to terpenes, abiotic stresses also induce the emission of other volatile compounds like C_6_ compounds, also called LOX-products (Loreto and Schnitzler, 2010). C_6_ compounds derive from polyunsaturated fatty acids (linolenic and linoleic acids) and are responsible for green aroma in grape berries. They are emitted due to membrane denaturation and damage by lipoxygenation (Capitani et al., 2009). One of the first recognized ozone effects is the denaturation of the lipids in cellular membranes (Pell et al., 1997). Therefore, these volatiles associated with lipid peroxidation could be increased in ozone-stressed grapes. In addition, as a result of the cell structure changes that dramatically occur during grape dehydration, lipoxygenase is released. This oxidative enzyme is involved in lipid oxidation and facilitates the production of C_6_ alcohols and aldehydes (Moreno and Peinado, 2012).

In our work, hexanol was the only free C_6_ compound identified. Ozone induced a non-significant (*p* > 0.01) decrease of free hexanol in fresh and withered grapes, which was stronger in withered grapes after ozone treatment at normal dose. Other studies demonstrated that the combined effect of postharvest dehydration and ozone exposure negatively influenced free hexanol contents at the beginning of grape dehydration (5% weight loss) but the biosynthesis was induced at the last stages of dehydration (30% weight loss) through the lipoxygenase-hydroperoxide lyase (LOX-HPL) pathway (Río Segade et al., 2017). In the present work, short-term ozone treatment also seems to elicit the synthesis of bound hexanol in fresh grapes when they were treated for 48 h particularly at the high ozone dose. However, the content of bound hexanol was not affected by ozone in withered grapes.

## Conclusion

In wine industry, ozone is useful for the management of grape microbiota and may also be interesting for its positive effects in alternative grape processing systems on secondary metabolites, such as VOCs. This study provides new knowledge on the single effect of oxidative stress caused by the ozone application in postharvest short-term treatment and the combined effect of abiotic stresses, namely oxidative and dehydration, on Moscato bianco volatile compounds. The results obtained highlighted that glycosylated and free VOCs responded differently to grape ozone exposure in all the samples analyzed. In fresh grapes, the content of total free VOCs decreased significantly in ozone-treated samples mainly due to the oxidative processes caused by ozone that negatively affected linalool and neral among terpenes, while glycosylated VOCs exhibited a trend to increase their concentration particularly at the higher ozone dose (60 μL/L for 24 and 48 h). This increase induced by ozone of glycosylated forms is essential for wine quality because the corresponding free forms will be released in the wine during fermentation by glycosidase activities from yeasts.

Partial postharvest grape dehydration is a technique traditionally used for sweet and fortified wines production. However, it usually causes the decrease of VOCs content, mainly terpenes, in the free fraction, which greatly contributes to the aroma of Muscat grape varieties. Postharvest short-term ozone treatment reduced the volatile loss produced by dehydration, preserving the Moscato bianco aromatic profile. Particularly the use of ozone at 60 μL/L for 48 h better preserved free linalool, *cis*-furan linalool oxide, and α-terpineol, and even could have induced the synthesis of free (+)-4-carene and 4-terpineol, with respect to control. The double abiotic stress response, specifically short-term ozone treatment followed by partial dehydration, could be an innovative strategy to improve the grape aromatic profile and therefore the wine quality when the dehydration process is necessary to produce special wines.

This work can contribute to better understand how different grape metabolic compounds respond to various abiotic stresses, applied alone or in combination, as well as the possible interaction among them.

## Author Contributions

LR designed the research. SRS and SG conducted and controlled the experiments. MP and FT determined volatile compounds. MV analyzed data. MV and SRS wrote the manuscript. All authors contributed to editing the manuscript.

## Conflict of Interest Statement

The authors declare that the research was conducted in the absence of any commercial or financial relationships that could be construed as a potential conflict of interest.

## Footnotes

1 http://webbook.nist.gov/chemistry/
